# Lymphadenitis as a Rare Side Effect of H1N1 Vaccine in a Child

**DOI:** 10.1155/2010/459543

**Published:** 2010-12-06

**Authors:** Zuhal Gundogdu, Mualla Seyhogullari

**Affiliations:** Kocaeli Medical Centre, 41100 Kocaeli, Turkey

## Abstract

We present a 5-year-old boy who had the complaint of swelling and pain on the right vaccine shot and right axillary areas. The right axillary area was diagnosed as reactive lymphadenitis, which we believe is a rare local side effect of the swine flu vaccine. The key message to take away from this case is that the patient had lymphadenitis as a local side effect of the swine flu vaccine. Lymphadenitis should be reported as a possible local side effect of the swine flu vaccine.

## 1. Introduction

2009 H1N1 influenza also called Swine Flu, is caused by a new strain of the influenza virus, and it has spread through many countries. Vaccines are available to protect against 2009 H1N1 influenza a vaccine, like any medicine, could cause serious problems such as a severe allergic reaction. However, the risk of any vaccine causing serious harm, or death, is extremely small [1]. 

Approximately 44% of people who were administered swine flu vaccine reported mild side effects within 7 days of receiving the first dose of swine flu vaccine of CSL Biotherapies. 2.5% of the vaccine recipients reported yet moderate local side effects, and there were no severe adverse events reported following immunization [2]. 

Lymphadenitis is the inflammation and/or enlargement of a lymph node. The most common symptoms of lymphadenitis are swelling of one or more lymph nodes which may feel slightly hardened and may be painful when touched. 

## 2. The Case Study

On December 9, 2009, a previously healthy 5-year-old boy with no history of illness was brought into the paediatrics clinic with complaints of pain in the upper part of the right arm (vaccine shot area), accompanied by swelling, bruising, and pain in the right axillary without any sign of fever. His past medical history, family history, and social history are unremarkable. 

He had swine flu vaccine administered intramuscularly in a local health authority clinic on the 8th of December 2009. Novartis H1N1 vaccine was administered to the child with a signed parental consent form requested by the Turkish Ministry of Health. Parents of the boy realised a swelling in the right arm where the vaccine was administered as well as a swelling in the right axillary area on the night of the same day when the boy started to complain about a pain. When he was brought into the paediatrics clinic on December 9, 2009, a day after the vaccination, examination revealed a hard and painful mass of nearly 2 cm in diameter on the right upper arm shot area with two other painful but small right axillary swellings. The examination of the other systems revealed no pathology. For the differential diagnosis, axillary ultrasonography (USG) examination was requested alongside tests for blood count (CBC) and C-reactive protein (CRP). Laboratory investigations of CBC and CRP were normal. The patient was not given any anti-inflammatory, antiallergic or antibiotic treatments. 

The boy was called into the clinic three days later on the 12th of December 2009 for a physical examination; it was found that swelling of the vaccine shot area still existed although the pain had decreased. The parents turned down our request to take a biopsy for histopathological examination.

Ultrasound is a useful imaging modality in assessment of lymph nodes, and its features can help only in identifying abnormal nodes including size, shape, echogenic hilus, hypoechogenicity or isoechogenicity, echogeneity, coagulation necrosis, and a sharp nodal border [3]. 

Ultrasound features can help identify whether lymphadenitis is reactive or not. In this case, three USG results showed that this is a postvaccine lymphadenitis because of long-to-short-axis ratio (L/S ratio), oval shape, and hyperechoic hilum.

**Figure 1:**
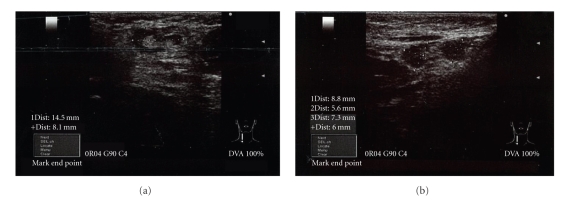
USG scans of the right axillary area taken one day after the vaccination on the 9th of December 2009.

A right axillary USG showed a reactive lymph nodule in the superior area with a size of 15 × 8 mm (Figure 1(a)) and two in the inferior area with dimensions of 9 × 6 mm and 7 × 6 mm (Figure 1(b)). They were of fuzzy form shape with hypoechogenic cortex with clear echogenic hilus indicator. In the Doppler mode, only hilus vascular structures were observed. These findings are meaningful for acute reactive lymphadenitis. Fuzzy form and echogenic hilus indicate a benign lymphadenitis as hypoechogenic cortex is usually observed in acute cases. 

**Figure 2:**
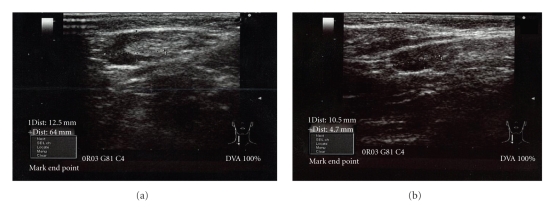
USG scans of the right axillary area taken 2 weeks after the vaccination on the 22nd of December 2009.

The boy had been called into the outpatients' clinic a week later on the 16th of December 2009 when a second USG taken did reveal that 7 × 6 mm nodule had disappeared and there were no significant changes in the structure and size of the other two nodules. Bruising had completely disappeared. However, the third USG scan carried out two weeks later on the 22nd of December 2009 revealed a decrease in nodule dimensions from 15 × 8 mm to 12 × 6 mm (Figure 2(a)) and the second nodule changed from 9 × 6 to 10 × 4 mm as shown in Figure 2(b). Furthermore, a decrease in the cortex echo and increased hilus echogeneity were also observed indicating reactive acute lymphadenitis. The physical examination carried out on the fifteenth day revealed that the patient's arm was back to normal.

## 3. Discussion

86% of the volunteers who received Novartis's H1N1 (7.5 *μ*g of MF59 adjuvanted) vaccine reported adverse reactions after one or both doses—the most common local side effect experienced was a pain in the injection site. The reactions were generally mild or moderate and resolved themselves after 72 hours [4]. The majority of reported local adverse events, or side effects occurring at the location where either vaccine had been administered, include tenderness pain, redness, hardening of skin, swelling, and bruising [2, 4]. The boy presented in this report started complaining about pain and swelling on the night of the same day he had received H1N1 vaccination. 

Systemic effects were also reported by CSL Biotherapies and Novartis vaccine recipients. Approximately 36% of volunteers who received the swine flu vaccine manufactured by CSL experienced mild systemic side effects. 8% of the vaccine recipients reported moderate systemic side effects, and less than 1% experienced a severe adverse reaction to immunization. Severe side effects reported include malaise, muscle pain, and nausea. Muscle aches were the most common systemic side effect reported by participants receiving the H1N1 vaccine produced by Novartis, and no severe systemic side effects were reported. The following are common whole-body side effects occurring in response to either H1N1 vaccination: headache, malaise, muscle pain, chills, nausea, fever, and vomiting. In addition, researchers evaluated the occurrence of select neurological adverse events [2, 4]. 

When a lymph node rapidly increases in size, its capsule stretches and causes pain. Pain is usually the result of an inflammatory process [3]. Most inflammatory diseases involve lymph nodes diffusely and homogeneously, generally preserving their oval shape. If threshold value of the long-to-short-axis ratio (L/S ratio) employed is low, then the accuracy of US in differentiating normal/reactive node (oval shape) from pathogic nodes (rounded shape) is also relatively low (sensitivity 71%, specifity 65%) [5]. If the ratio used is 2.0, sensitivity increases to 81–95% and specifity to 67–96%. The second parameter to be assessed is the hyper echoic central line of lymph nodes (the hilum). The sonographic detection of the hyper echoic hilum has always been related to the probable benign nature of the lymph node [5]. 

Postvaccinal lymphadenitis is a reactive response of the lymph node to the vaccination. Some of the vaccines such as BCG, varicella zoster, and pneoumococcal can cause reactive lymphadenitis, and it is a rather common complication of BCG vaccination [6, 7]. Local reactions (generally erythema and induration with or without tenderness) are common after the administration of vaccines containing diphtheria, tetanus, or pertussis antigens. Occasionally, a nodule may be palpable at the injection site of adsorbed products for several weeks [8]. 

We followed this patient for nearly twenty days but we did not encounter any systemic reaction. This was not a suppurative lymphadenitis characterized by appearance of fluctuation with erythema and edema of the overlying skin. The size of lymphadenitis has decreased and disappeared completely after 15 days. 

We are of the opinion that further study is required for this type of side effect and a detailed histopathological examination could be of use. The parents turned down our request to take a biopsy for histopathological examination. However, our results have been conclusive enough that this case was a reactive lymphadenitis.

In a conclusion, reactive lymphadenitis is an unusual side effect of swine flu vaccination requiring further study, and to the best of our knowledge no cases in children have previously been reported in the literature neither by CDC nor by WHO sources, apart from a case reported in an adult [9].
